# mHealth Intervention to Promote Physical Activity Among Employees Using a Deep Learning Model for Passive Monitoring of Depression and Anxiety: Single-Arm Feasibility Trial

**DOI:** 10.2196/51334

**Published:** 2023-11-17

**Authors:** Kazuhiro Watanabe, Shoichi Okusa, Mitsuhiro Sato, Hideki Miura, Masahiro Morimoto, Akizumi Tsutsumi

**Affiliations:** 1 Department of Public Health Kitasato University School of Medicine Sagamihara Japan; 2 TOSHIBA Health Insurance Society Kawasaki Japan; 3 Health & Productivity Management Promotion Division Fujitsu General Limited Kawasaki Japan; 4 Nikkei Business Publications, Inc Tokyo Japan; 5 MS & AD InterRisk Research & Consulting, Inc Tokyo Japan

**Keywords:** eHealth, behavioral change, mobile phone, smartphone, mHealth, mobile health, app, apps, applications, monitor, monitoring, physical activity, exercise, fitness, application, workplace, distress, depression, depressive, anxiety, mental health, worker, workers, employee, employees, occupational health, satisfaction, feasibility, acceptability

## Abstract

**Background:**

Physical activity effectively prevents depression and anxiety. Although mobile health (mHealth) technologies offer promising results in promoting physical activity and improving mental health, conflicting evidence exists on their effectiveness, and employees face barriers to using mHealth services. To address these problems, we recently developed a smartphone app named ASHARE to prevent depression and anxiety in the working population; it uses a deep learning model for passive monitoring of depression and anxiety from information about physical activity.

**Objective:**

This study aimed to preliminarily investigate (1) the effectiveness of the developed app in improving physical activity and reducing depression and anxiety and (2) the app’s implementation outcomes (ie, its acceptability, appropriateness, feasibility, satisfaction, and potential harm).

**Methods:**

We conducted a single-arm interventional study. From March to April 2023, employees aged ≥18 years who were not absent were recruited. The participants were asked to install and use the app for 1 month. The ideal usage of the app was for the participants to take about 5 minutes every day to open the app, check the physical activity patterns and results of an estimated score of psychological distress, and increase their physical activity. Self-reported physical activity (using the Global Physical Activity Questionnaire, version 2) and psychological distress (using the 6-item Kessler Psychological Distress Scale) were measured at baseline and after 1 month. The duration of physical activity was also recorded digitally. Paired *t* tests (two-tailed) and chi-square tests were performed to evaluate changes in these variables. Implementation Outcome Scales for Digital Mental Health were also measured for acceptability, appropriateness, feasibility, satisfaction, and harm. These average scores were assessed by comparing them with those reported in previous studies.

**Results:**

This study included 24 employees. On average, the app was used for 12.54 days (44.8% of this study’s period). After using the app, no significant change was observed in physical activity (–12.59 metabolic equivalent hours per week, *P*=.31) or psychological distress (–0.43 metabolic equivalent hours per week, *P*=.93). However, the number of participants with severe psychological distress decreased significantly (*P*=.01). The digitally recorded duration of physical activity increased during the intervention period (+0.60 minutes per day, *P*=.08). The scores for acceptability, appropriateness, and satisfaction were lower than those in previous mHealth studies, whereas those for feasibility and harm were better.

**Conclusions:**

The ASHARE app was insufficient in promoting physical activity or improving psychological distress. At this stage, the app has many issues that are to be addressed in terms of both implementation and effectiveness. The main reason for this low effectiveness might be the poor evaluation of the implementation outcomes by app users. Improving acceptability, appropriateness, and satisfaction are identified as key issues to be addressed in future implementation.

**Trial Registration:**

University Hospital Medical Information Network Clinical Trials Registry UMIN000050430; https://tinyurl.com/mrx5ntcmrecptno=R000057438

## Introduction

### Background

Depression and anxiety are the most prevalent mental health conditions experienced by employees. In 2019, an estimated 301 million working-age adults reported living with anxiety, while approximately 280 million were affected by depression [1]. Additionally, numerous employees exhibited subclinical levels of depression, anxiety, and psychological distress [2-4]. These mental health conditions detrimentally impact individuals' quality of life and well-being, leading to increased instances of sick leave [5]. Moreover, the annual global economic cost attributed to mental health issues is estimated to reach approximately USD 1 trillion, primarily due to reduced productivity [1,6,7]. Considering the severity continuum, universal interventions and preventive strategies, which include healthy employees are crucial in addressing depression and anxiety within the realm of occupational health.

Engaging in physical activity is a vital health behavior with the potential to prevent the occurrence of depression and anxiety [8]. Its effectiveness has been established specifically within the working population [9]. Notably, it is among the most conspicuous universal approaches to proactively prevent the development of common mental disorders in the workplace [10]. Given the widespread prevalence of both physical inactivity and depression or anxiety among employees [11,12], promoting physical activity is a central focus in preventive initiatives addressing mental health conditions among employees.

Mobile health (mHealth) technologies offer promising results in promoting physical activity. The effective use of mHealth has the potential to increase access to evidence-based interventions, better inform consumers of care, and more actively engage them in treatment [13]. As yet, many trials have investigated the effectiveness of mHealth technologies such as SMS text messages, websites, wearable monitors, and smartphone apps to promote physical activity [14-16]. Behavior change techniques used in these interventions include feedback, goal setting, competition, social sharing, and reward [17]. In the working population, tailored SMS text messages combined with self-monitoring [18] and a gamified smartphone app based on the self-determination theory [19] were investigated in randomized controlled trials and reported significant increases in physical activity.

However, scientific evidence for the effectiveness of mHealth interventions in promoting physical activity is inconsistent [17,20]. Moreover, these interventions were not necessarily preventive for depression, anxiety, or stress-related indicators [21-23]. When stratifying physical activity into domain-specific activities, occupational and transport activities have weaker positive correlations with mental health outcomes than leisure-time activities [24]. Therefore, mHealth interventions that monotonically increase physical activity could not be effective in improving depression and anxiety. Moreover, employees face several barriers to using mHealth services and subsequently tend to discontinue the services [19]. In a previous study, although employees agreed that physical activity was effective in improving mental health and stated the need for individualized mHealth services, they also perceived limited effects of nonleisure physical activity and a lower priority for physical activity compared to sleep and rest [25]. To improve depression and anxiety by promoting physical activity among employees, mHealth services should consider working conditions and focus on leisure-time physical activity.

To address these problems, we recently developed a smartphone app named ASHARE to promote physical activity and prevent depression and anxiety in the working population. The app has functions based on basic behavior change techniques: self-monitoring, feedback, and data sharing of users’ physical activity. Moreover, the app implements a deep learning model for passive monitoring of depression and anxiety [26]. Briefly, this model predicts the level of depression and anxiety from the previous day’s physical activity patterns obtained via the Google Fit app, working conditions (occupation, employment status, work shift types, and working hours), and demographics. Owing to the model, feedback and comments on physical activity and mental health were provided automatically to users without the effort associated with inputting their mental health status. In addition, the model may have an advantage in helping the users find a relationship between physical activity and mental health. Furthermore, the feedback and comments can be customized according to the working conditions and health status of the individual. For example, the model does not necessarily consider high levels of physical activity as a predictor of good mental health, and advises on rest when long working hours are input. This could contribute to the high adoption rate of the app and its high effectiveness in the prevention of mental health conditions. However, it is uncertain whether the developed app and the deep learning model are effective in improving physical activity and depression and anxiety. In addition, at this stage, the implementation of the app, that is, acceptability, appropriateness, feasibility, satisfaction, and potential harm are also unknown.

### Objectives

This study aimed to preliminarily investigate the effectiveness of ASHARE by employing a deep learning model based on physical activity to monitor depression and anxiety. This study also evaluated specific implementation outcomes—acceptability, appropriateness, feasibility, satisfaction, and potential harm caused by the app—and compared them with those reported in previous mHealth studies. We hypothesized that physical activity and psychological distress would significantly improve after using the app.

## Methods

### Study Design and Setting

This study was a 1-month single-arm feasibility trial. From March to April 2023, we (KW, SO, MS, HM, and MM) recruited employees via snowball sampling. A web-based announcement about this study was made to these employees. These employees were asked to complete the web-based baseline survey via a URL sent by the research team, install the ASHARE app on their private smartphones, and use it for 1 month. During the intervention period, log data from the app, including when and how many times the participants logged in and the duration of physical activity (minutes per day), were stored and used in the analyses. After the 1-month period, the research team sent the URL of a web-based follow-up survey to the participants to test the implementation and effectiveness of the intervention.

This study’s protocol was registered in the University Hospital Medical Information Network Clinical Trials Registry (UMIN000050430). This paper was reported in accordance with the guidelines for nonrandomized evaluations of behavioral and public health interventions—the TREND (Transparent Reporting of Evaluations with Nonrandomized Designs) statement [27].

### Participants

This feasibility trial included employees who (1) were aged 18 years or older, (2) could answer questionnaires written in Japanese, and (3) had a smartphone for personal use. This study excluded those who (1) were currently absent or (2) had been absent in the past 12 months.

### Interventions

#### Contents and Functions of the App

**Figure 1:**
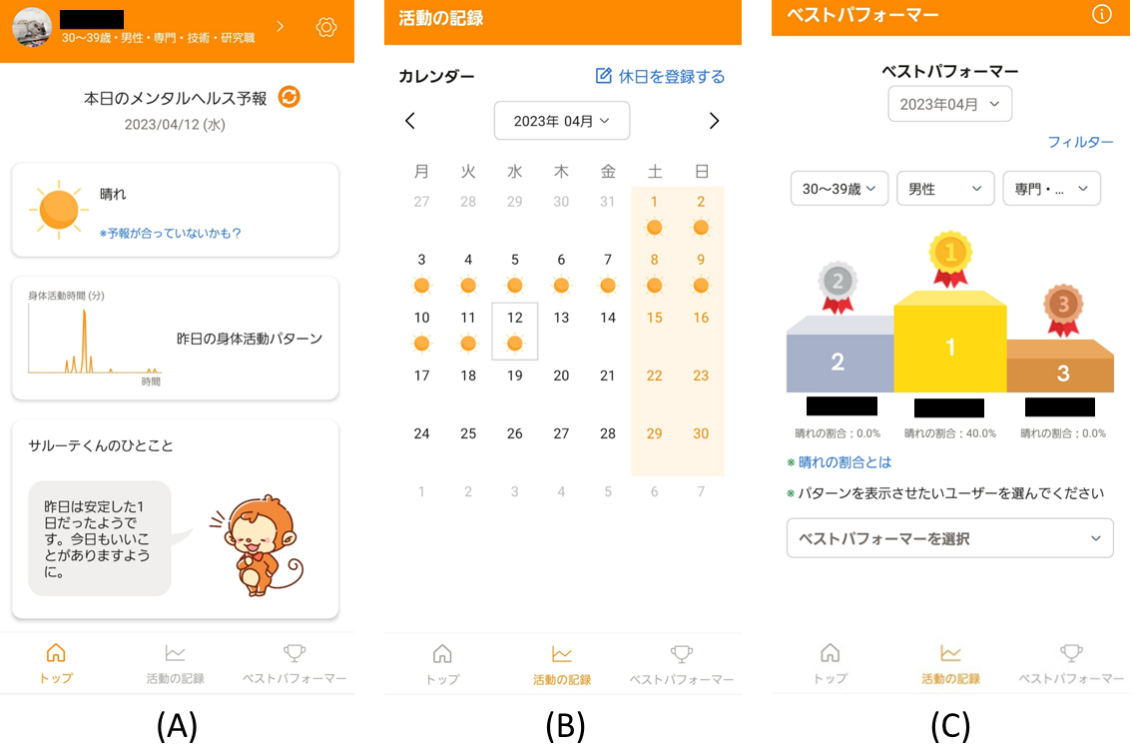
The interface of the smartphone app ASHARE: (A) top screen including feedback on physical activity and mental health forecast; (B) a record of mental health forecast to monitor them on the monthly calendar; (C) “best performer”: users with a high percentage of sunny days.

The participants included in this study installed the ASHARE app on their private smartphones and used it for 1 month. ASHARE is a native app for iOS and Android smartphones, supporting version 12.0 or later in iOS, and version 5.0 or later in Android. The iOS and Android versions of the app were distributed via the TestFlight and DeployGate testing environments, respectively. The app was developed based on basic behavior change techniques, namely, self-monitoring, feedback, and sharing of users’ physical activity data. Moreover, the needs of the employees identified in the qualitative interviews were reflected in the functions and interface of the app [25]. Figure 1 presents the ASHARE interface.

After installing the app, a user was initially asked to register their username and email address and input their age, gender, occupation, employment status, shift type, working hours per week, holiday patterns, and preferred activities. The app works with Apple Healthcare (iOS) and Google Fit (Android) and obtains the duration of moderate to vigorous physical activity (MVPA) every 15 minutes [28]. These data are stored on a cloud server. The duration of the overall physical activity on the previous day is depicted as a graph on the top screen of the app (Figure 1A). In addition, the deep learning model accesses the data on the previous day on cloud servers when the user starts the app and automatically predicts a score of psychological distress of the day measured using the 6-item Kessler Psychological Distress Scale (K6) [29]. Based on the K6 cutoff scores [30,31], participants were provided with feedback using predicted scores that are presented as weather conditions accompanied by rule-based comments from a monkey anime character (mental health forecast): sunny as light level (<5), cloudy as subthreshold level (≥5 and <13), and rainy as severe level (≥13) of distress (Figure 1A). Using the notification function on the smartphone, the mental health forecast was provided at 6 o'clock every morning. The predicted levels of psychological distress are recorded and monitored on a monthly basis (Figure 1B). As a function of data sharing, users with a high percentage of sunny days are ranked as “best performers” (Figure 1C). Rankings can be modified freely based on categories such as age, gender, occupation, and preferred activities. If users are reluctant to share data with other users, they can decline to participate in the ranking process.

#### Requirements for the Participants During the Intervention Period

No requirement was made for the participants to use the app during the intervention period. Except for the initial registration of the demographics and working conditions, all feedback and comments were automatically recorded. The participants could open the app at any time. The ideal usage of the app, as expected by the researchers, was for the participants to take about 5 minutes every day to open the app, check their physical activity patterns and the results of their mental health forecast, and increase the amount of any type of MVPA.

#### Deep Learning Model Implemented in the App

The deep learning model implemented in the app was the LSTM model [26], which is one of the recurrent neural network models. The length of the input was set to 96, which was equal to the length of the data on the physical activity for a single day (24 hours for every 15 minutes) obtained by Apple Healthcare and Google Fit. The registered information about age, gender, occupation, employment status, shift type, working hours per week, holiday patterns, and predicted scores of K6 on the day were also used and expanded to 96 lengths to align with the length of the physical activity. Categorical variables were one-hot encoded. The output was a log-transformed score on the K6 scale on the next day. The number of hidden layers in the LSTM model was 27. Regarding the activation functions, the rectified linear unit, sigmoid, and linear activations were used as the hidden, recurrent, and output layers, respectively. A previous study on the predictive performance of the deep learning model underlying the ASHARE app reported that the correlation coefficient between the predicted and measured values for psychological distress was 0.679 (*R*^2^=0.463) and that the overall classification accuracy for levels of psychological distress was 76.3% [26].

### Outcomes

#### Physical Activity

The primary outcome measure was the total amount of physical activity. The Japanese version of the Global Physical Activity Questionnaire (GPAQ version 2) was used to measure physical activity [32]. This scale is widely used to assess the level of physical activity in epidemiologic studies and has been demonstrated to have acceptable reliability and convergent validity in 9 countries, including Japan [33]. Metabolic equivalents (METs) were used as the unit of physical activity intensity. The amount of physical activity per week (MET hours per week) was calculated as a continuous variable according to the GPAQ analysis guide [34]. Overall physical activity levels were assessed as ordinal variables in 3 levels (low, moderate, and high).

The duration of physical activity (minutes per day) was also used as an objective measure. This information was obtained from the cloud server of the app. Apple Healthcare (iOS) and Google Fit (Android) recorded MVPA time every 15 minutes. We aggregated the values as units per day. The reliability and validity of the measurement of activities in these smartphone apps were confirmed in several populations [35-37].

#### Psychological Distress

As a secondary outcome, self-reported psychological distress via K6 [29] was used as an indicator of depression and anxiety, which was consistent with the predictions of the deep learning model. K6 consists of 6 items that assess how often people experience symptoms of depression and anxiety; these items were rated on a 5-point Likert scale (0=none of the time and 4=all the time). The validity of the Japanese K6 scale has been previously confirmed [30], while the internal consistency was found to be quite high in this study (Cronbach α=.92). The total score on the K6 scale was calculated, and the participants were divided into 3 groups based on the K6 cutoff scores: mild (<5), subthreshold (≥5 and <13), and severe (≥13) [30,31].

#### Implementation Outcomes

The Implementation Outcome Scale of Digital Mental Health (iOSDMH) for users was used [38]. The iOSDMH for users has a total of 19 items, consisting of acceptability (3 items), appropriateness (4 items), feasibility (6 items), satisfaction (1 item), and harm (5 items). Internal consistency, structural validity, and known-class validity were confirmed in the scale development study and a previous systematic review [38,39]. We calculated the total scores of each subscale, and the overall score comprised acceptability, appropriateness, feasibility, and satisfaction.

As an objective measure of the app's implementation, the number of days logged in during the intervention period was obtained from recorded data on the cloud server.

### Sample Size Calculation

As this study was a feasibility trial, this study’s protocol required that we recruit a small sample in advance, and thus, an a priori sample size calculation was not conducted. Instead, we calculated the effect sizes (Cohen *d*) of the intervention on the continuous outcomes, and the statistical power (1–β) was calculated ad hoc using G*Power (version 3.1.9.2; Heinrich-Heine-Universität Düsseldorf) [40].

### Statistical Analysis

The participants’ demographic characteristics were summarized. To investigate the effectiveness of the app, descriptive statistics (frequencies, means, and SDs) of the primary and secondary outcomes were summarized at baseline and at the 1-month follow-up. Statistical tests for score changes were conducted in complete cases: paired *t* test for continuous outcomes, and chi-square test for categorical outcomes. For continuous outcomes, the effect sizes (Cohen *d*) for score changes were calculated. Mixed modeling was conducted to estimate a linear change in the duration of physical activity (minutes per day) from the installation of the app (baseline) to the 28th day. We estimated the time effect of the change using random effects for the intercept and time. As potential modifiers for the effect, the operating system in personal smartphones (iOS [reference] and Android), age (20-30 years, 40-50 years [reference], and 60 years or older), gender (male [reference], female), and the total K6 score at baseline were entered into the model as covariates. Mean scores of implementation outcomes based on iOSDMH for users were compared with those reported in previous mHealth studies [39]. All analyses were conducted at the individual level using SPSS Statistics (version 25.0, IBM Corp).

### Ethical Considerations

This study’s protocol was approved by the Kitasato University Medical Ethics Organization (C22-137). Informed consent was obtained from all participants before the baseline survey. Potential participants who accessed the URL for the baseline survey were asked to read and approve the terms and conditions of this study before proceeding to the actual survey. The terms and conditions stated that the research team would protect the privacy and confidentiality of the participants, that this study data would be deidentified before analyses, that participants could withdraw from this study at any time without stating reasons, and that withdrawal would not result in any disadvantages to them. As an incentive, the participants received an Amazon gift certificate worth JPY 3000 (US $22.2 in the conversion rate as of March 2023) when they completed the baseline survey.

## Results

### Characteristics of the Participants

**Figure 2:**
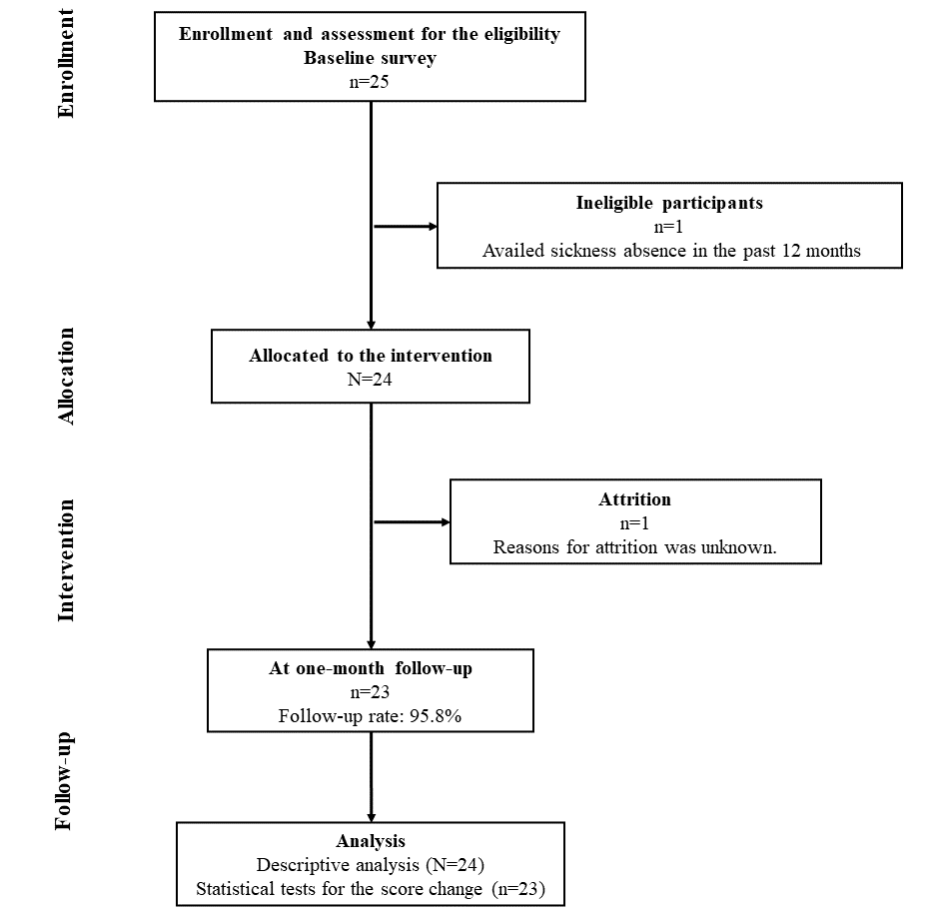
Participant flowchart.

Figure 2 presents the flowchart of this study. After recruitment, 25 employees accessed and completed the baseline questionnaire between March and April 2023. After screening for eligibility, 1 participant who had availed sickness absence in the past 12 months was excluded, and finally, 24 participants were included in this study. At the 1-month follow-up, 23 of the 24 (95.8%) participants completed the questionnaire between April to May 2023. It is unknown why the participant dropped out at the follow-up stage. This participant had reported extremely high levels of physical activity (224.0 MET-h) and severe psychological distress (K6 total score=15).

Table 1 shows the baseline demographic characteristics of the participants. A total of 14 (58.3%) female and 10 male (41.7%) employees joined this study. Over 80% (n=20) were full-time employees, and all participants worked daytime shifts. The majority of the participants were clerks (45.8%, n=11), professionals, engineers, academics (25%, n=6), and managers (16.7%, n=4). The participants joined this study using iOS (70.8%, n=17) and Android (29.2%, n=7) personal smartphones.

**Table 1:** Participants’ characteristics at the baseline (N=24).

Variables			Participants, n (%)
**Age (years)**			
	20-29	2 (8.3)	
	30-39	5 (20.8)	
	40-49	7 (29.2)	
	50-59	6 (25)	
	60-69	4 (16.7)	
**Gender**			
	Female	14 (58.3)	
	Male	10 (41.7)	
	Not to say or others	0 (0)	
**Operating system (OS) in smartphone**			
	iOS	17 (70.8)	
	Android	7 (29.2)	
**Employment status**			
	Full-time	20 (83.3)	
	Contract or dispatched	3 (12.5)	
	Others	1 (4.2)	
**Shift type**			
	Day shift	24 (100)	
**Occupation**			
	Managers	4 (16.7)	
	Professionals, engineers, or academics	6 (25)	
	Clerks	11 (45.8)	
	Services	1 (4.2)	
	Others	2 (8.3)	
**Working hours (per week; hours)**			
	1-34	1 (4.2)	
	35-40	5 (20.8)	
	41-50	13 (54.2)	
	51-60	2 (8.3)	
	61-65	0 (0)	
	66-70	2 (8.3)	
	71 or more	1 (4.2)	

### Effects of the Intervention Program on the Outcomes

Table 2 presents the descriptive statistics for physical activity and psychological distress at baseline and 1-month follow-up. At baseline, the total amount of physical activity was 36.60 MET hours per week, and 20.8% (n=5) of the participants ensured a high level of physical activity according to the GPAQ standard. After 1 month of use, the amount of physical activity and the number of participants who ensured a high level of physical activity decreased to 24.01 MET hours per week (*P*=.31) and 4.3% (n=1, *P*=.31), respectively. In contrast, the duration of physical activity recorded by the app increased by 11.06 minutes per day after using the app (*P*=.48). The effect sizes (Cohen *d*) on these outcomes were –0.38 (95% CI, –0.96 to 0.20) and 0.23 (95% CI, –0.41 to 0.88). The mean score of psychological distress was 4.13 (SD 4.5) at the baseline, and 12.5% (n=3) of the participants reported severe psychological distress at baseline. After using the app, the mean distress score decreased to 3.70 (SD 3.6; *P*=.93), and the number of participants with severe psychological distress decreased significantly (*P*=.01). Cohen *d* on the mean change for psychological distress was –0.11 (95% CI, –0.68 to 0.47). The statistical powers (1–β) calculated ad hoc for the amount and duration of physical activity and psychological distress were 0.41, 0.15, and 0.08, respectively.

**Figure 3:**
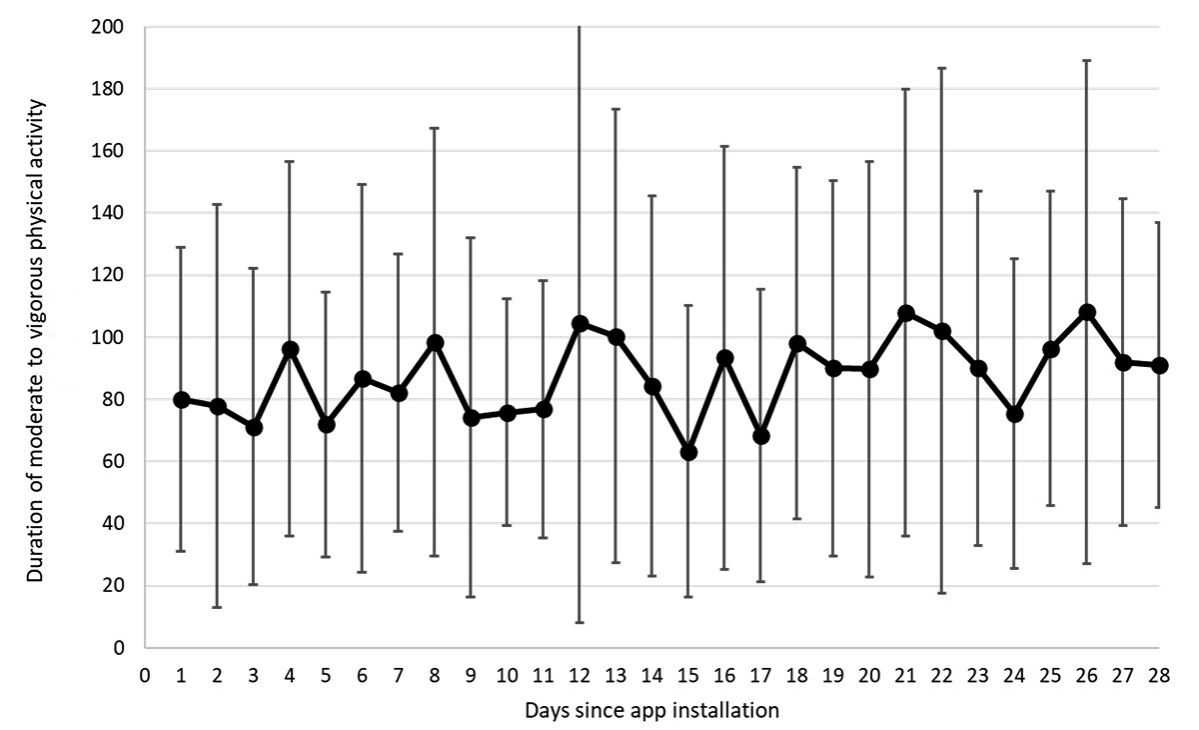
Duration of moderate to vigorous physical activity (MVPA) since app installation (minutes per day).

Figure 3 shows the mean duration of MVPA (minutes per day) recorded by the app from the installation of the app to the 28th day. Following the mixed modeling approach, the linear effect of the MVPA time was found to be positive and marginally significant (+0.60 minutes per day, *P*=.08), even after entering the covariates, namely the OS of the smartphones, age, gender, and psychological distress at baseline.

**Table 2:** Physical activity and psychological distress at the baseline and 1-month follow-up (N=24).

		Baseline		1-month follow-up		Cohen *d* (95% CI)	*P* value for change	
		n (%)	Missing n (%)	n (%)	Missing n (%)			
Physical activity as total amount of physical activity (MET^a^ hours per week)^b^		N/A^c^	0 (0)	N/A	1 (4.2)	–0.38 (–0.96 to 0.20)	.31	
**Level of physical activity**								.31
	High^d^	5 (20.8)	0 (0)	1 (4.3)	1 (4.2)	—^e^		
	Moderate^f^	15 (62.5)	0 (0)	13 (56.5)	1 (4.2)	—		
	Low^g^	4 (16.7)	0 (0)	9 (39.1)	1 (4.2)	—		
Duration of physical activity (minutes per day)^h^		N/A	5 (20.8)	N/A	6 (25)	0.23 (–0.41 to 0.88)	.48	
Psychological distress: total K6^i^ score^j^		N/A	0 (0.0)	N/A	1 (4.2)	–0.11 (–0.68 to 0.47)	.93	
**K6 classification**								
	Light (K6 ≤ 4)	16 (66.7)	0 (0)	16 (69.6)	1 (4.2)	—	.01	
	Subthreshold (5 ≤ K6 ≤ 12)	5 (20.8)	0 (0)	7 (30.4)	1 (4.2)	—		
	Severe (13 ≤ K6)	3 (12.5)	0 (0)	0 (0)	1 (4.2)	—		

^a^MET: metabolic equivalent.

^b^Physical activity was 36.6 (SD 42.8) MET hours per week at baseline and 24.01 (SD 18.7) MET hours per week at 1-month follow-up.

^c^N/A: not applicable.

^d^High: ≥3 days of vigorous-intensity activity with ≥1500 MET minutes per week or ≥7 days of any combination of walking or moderate to vigorous physical activity with 3000 metabolic equivalent minutes per week.

^e^Not available.

^f^Moderate: ≥3 days of vigorous-intensity activity with ≥20 minutes per day or ≥5 days of moderate-intensity activity or walking with ≥30 minutes per day or ≥5 days of any combination of walking or moderate- or vigorous-intensity activities with ≥ 600 metabolic equivalent minutes per week.

^g^Low: not meeting the criteria of high or moderate physical activity.

^h^Physical activity duration was 80.05 (SD 48.9) minutes per day at baseline and 91.11 (SD 45.9) minutes per day at 1-month follow-up.

^i^K6: 6-item Kessler Psychological Distress Scale.

^j^K6 score was 4.13 (4.5) at baseline and 3.70 (SD 3.6) at 1-month follow-up.

### Evaluation of the Implementation Outcomes

On average, the app was used for 12.54 (44.8%) days from installation to the 28th day. Usage days per user ranged from 4 (14.3%) to 28 days (100%). Table 3 describes the mean scores of the implementation outcomes measured using iOSDMH. Compared to previous mHealth intervention studies, the evaluation of acceptability, appropriateness, and satisfaction were significantly lower in this study. The effect sizes (Cohen *d*) for the differences was –0.57 (95% CI, –0.99 to –0.16) for the overall score, and was large for satisfaction (–1.15, 95% CI, –1.56 to –0.73). However, the scores for feasibility and harm were better. A large difference was observed in the evaluation of harm (–0.78, 95% CI, –1.19 to –0.36).

**Table 3:** Implementation outcomes of the app.

	This study ASHARE (n=23), mean (SD)	Previous studies^a^ (6 studies, total N=1945), mean (SD)	*P* value for *t* test	Cohen *d* for difference (95% CI)
Overall score (14 items; 14-56)	36.65 (5.1)	40.62 (6.9)	<.001	–0.57 (–0.99 to –0.16)
Acceptability (3 items; 3-12)	6.87 (1.5)	8.38 (1.8)	<.001	–0.83 (–1.24 to –0.42)
Appropriateness (4 items; 4-16)	9.96 (2.3)	11.70 (2.4)	<.001	–0.73 (–1.14 to –0.31)
Feasibility (6 items; 6-24)	17.74 (2.8)	17.65 (3.2)	<.001	0.03 (–0.38 to 0.44)
Satisfaction (1 item; 1-4)	2.09 (0.7)	2.95 (0.75)	<.001	–1.15 (–1.56 to –0.73)
Harm (5 items; 5-20)	6.43 (1.6)	8.75 (3.0)	<.001	–0.78 (–1.19 to –0.36)

^a^The scores were reported in a previous systematic review [39].

## Discussion

Although the number of participants who had severe psychological distress decreased significantly, our hypotheses were not supported: the overall preventive effect of the app was not enough to promote physical activity or improve psychological distress. The main reason for this low effectiveness might be the poor evaluation of the implementation outcomes by app users. At this stage, this preliminary trial clarified that the developed app has many issues that are to be addressed in terms of both implementation and effectiveness.

Physical activity did not increase significantly, as revealed by the 1-month follow-up survey. The participants were already physically active at the baseline. A previous observational study reported that full-time employees spent approximately 45 minutes per day in MVPA, measured by a triaxial accelerometer [41]. Another study measured physical activity using the GPAQ and reported that approximately 10% of the participants were classified as having a high level of physical activity [42]. These reports support that the participants in this study might be an active group at baseline. This is a possible reason for the insignificant increase in physical activity. Furthermore, regression toward the mean might have occurred.

Contradictory results were observed for subjective and objective measurements of physical activity: physical activity measured using the GPAQ decreased, while that using the objective measure increased after using the app. The accuracy of self-reported measures is lower than that of pedometers or accelerometers, and it is possible for people to over-report or overestimate physical activity owing to social desirability bias [33]. Therefore, the GPAQ results were likely to contain more measurement bias and errors.

A significant decrease was observed in the proportion of participants with severe psychological distress after using the app. The app allowed users to passively monitor their levels of psychological distress by tracking their physical activity. Timely monitoring of mental health may provide feedback to users and lead to early detection of changes in depression and anxiety. This technique offered significant improvement in depressive symptoms and psychological health in a clinical setting [43], and is promising for the working population that has substantial levels of depression and anxiety. However, the overall effects of psychological distress remain unclear. Similar to the results for physical activity, regression toward the mean may have occurred in participants with severe levels of distress at baseline.

Contrary to our expectations, participants were not satisfied with the app, rated it as having poor acceptability and appropriateness, and did not use it as much. The decreasing trend in app use was similar to that observed in previous studies [19]. There are several possible reasons for the low ratings for the implementation outcomes. The main reason for this might be that the feedback content did not change significantly. The app provided users with only 3 types of feedback for psychological distress based on K6 cutoff scores (sunny, cloudy, and rainy). In this case, a user who maintains similar physical activity patterns might be offered feedback only for the same weather every day (Figure 1B), and thus, the app might not be able to grasp minute changes in depression and anxiety. Therefore, a modification of the app may be required to allow users to check the raw scores predicted by the deep learning model. Rule-based comments may reinforce users’ feelings of irreplaceable feedback and may not provide much input on how to change their behavior. In a previous mHealth study, reinforcement learning was implemented to select personalized feedback messages from a message pool [44,45]. Additional techniques could provide variety and offer more personalized messages.

Evaluations of feasibility and harm were favorable. Users were only required to register their demographic details and working conditions when they first used the app, and the recording of physical activity and predicting psychological distress were automated within the app. This feature can lead to ease of use and low effort when using the app.

This study has several limitations. First, as this was a single-arm feasibility trial, we cannot conclude that the changes in outcomes were due to app use. Many moderators exist, such as motivation for health promotion, job stress, and medical history. The statistical power of the change in outcomes was low, and the statistical analysis produced ambiguous results. Well-designed or randomized controlled trials are required in the future. Second, the data on the duration of physical activity on the app server had missing values. On the whole, of a total of 672 observational days (28 days × 24 participants), 116 days (17.3%) were missing values for the tracked physical activity. Since data were not recorded until the users opened the app on a particular day, records could not be obtained from participants who used the app infrequently. These participants may have been physically inactive and less motivated for the present intervention. Therefore, the measurement may include only information from the participants who were more active, and may have overestimated the activity time. Some participants might have had privacy concerns, even though the ASHARE app included a privacy policy [46]. In addition, substantial measurement errors and bias may be included in the objective MVPA in free-living conditions. Although previous studies reported the acceptable reliability and validity of Apple Healthcare and Google Fit, underestimation could occur when the participants do not carry the devices appropriately [47]. Third, the generalizability of this study was not high because all participants worked during the daytime, and most were full-time employees. Moreover, compared with the overall population of Japanese employees, the participants included more professionals, engineers, and academics. Other working populations, particularly night-shift employees, were not covered in this study.

In conclusion, the preventive effect of the developed app ASHARE was not enough to promote physical activity or reduce psychological distress. It remains unclear which mHealth interventions are feasible and effective in promoting physical activity and preventing depression and anxiety in healthy working populations. For the ASHARE app, improving acceptability, appropriateness, and satisfaction are identified as key issues to be addressed in future implementation.

## Abbreviations

GPAQ: Global Physical Activity Questionnaire
iOSDMH: Implementation Outcome Scale of Digital Mental Health
K6: 6-item Kessler Psychological Distress Scale
MET: metabolic equivalent
mHealth: mobile health
MVPA: moderate-to-vigorous physical activity
TREND: Transparent Reporting of Evaluations with Nonrandomized Designs

## Data Availability

The data sets generated and analyzed during this study are not publicly available because informed consent for public or secondary use has not been obtained from the participants; however, the data are available from the corresponding author upon reasonable request. The program codes in SPSS (.sps; IBM Corp) used in the statistical analyses are available in the Google Drive repository. Please contact the corresponding author if you want to access the program codes.
